# Editorial: Emerging paradigms in understanding cancer metastasis: focus on tumor microenvironment dynamics

**DOI:** 10.3389/fmolb.2025.1736325

**Published:** 2025-11-17

**Authors:** Imran Khan, Mohd Farhan, Hamidullah Khan, Mustafa Aziz Hatiboglu

**Affiliations:** 1 Department of Radiation Oncology, Mayo Clinic, Phoenix, AZ, United States; 2 Department of Oncology, Lombardi Comprehensive Cancer Center, Georgetown University Medical Center, Washington, DC, United States; 3 MedStar Health Research Institute, Washington, DC, United States; 4 Department of Molecular Biology, Beykoz Institute of Life Sciences and Biotechnology, Bezmialem Vakif University, Istanbul, Türkiye; 5 Department of Neurosurgery, Bezmialem Vakif University Medical School, Istanbul, Türkiye

**Keywords:** metastasis, liquid biospsy, post translation modification, targeted therapy, nanopartical

Cancer metastasis accounts for 90% of cancer-related deaths and is considered a crucial aspect posing a formidable challenge to the clinical management of patients. This Research Topic compiles five diverse studies exploring critical aspects of cancer metastasis.

Studies have identified the impact of non-histone lactylation (la) as a post-translational modification, playing a critical role in cancer metastasis by modulating the metabolism of metastatic cancer cells and precisely enhancing their immune evasion mechanisms. Jia et al. have highlighted the fact that higher lactylation profiles in the gastrointestinal tumors are associated with invasive tumors and poor clinical outcomes. Collectively, different studies have contributed to the identification of lactylation of several key proteins such as; Adenylate kinase 2 K28la, p53 lactylation at (K120 and K139), CBX3 K10la, DCBLD1 la, eEF1A2 K40 la, METT L3la, Nucleolin K477la, Cyclin E2la, ABCF1430la, YY1 K183la, ALDOA K230/K322la and PTBP1 K436la contribute to the metastatic progression of several cancer (Jia et al.). This compilation indicates a therapeutic window for targeting tumor metastasis via non-histone lactylation and motivates additional studies in this direction.

Determining the pathological spread of the primary tumors to the nearby lymph nodes is a key aspect in gastrointestinal cancer, specifically colon cancer (CC). The extent of disease spread to nearby lymph nodes critically influences the cancer staging and treatment strategy. In this regard, Aslan et al. demonstrated the effectiveness of the metastatic lymph node ratio (MLNR) in colon cancer patients, showing a significant association between MLNR and tumor recurrence in a retrospective study. The MLNR is a simple, independent prognostic marker for CC that could directly influence the CC staging in clinics. This study paved the platform for future prospective studies to validate the clinically applicable range of MLNR in cancer patients (Aslan et al.). Further, lymph node metastasis (LNM) significantly alters how we clinically handle cases of esophageal cancer (EC). The prognosis worsens, and this creates a fundamental difference in approaches involving neoadjuvant and surgical interventions. Nonetheless, as Wu et al. summarize, the currently available imaging and serologic techniques do not accurately identify nodal spread. Wu et al. merge several streams of nascent molecular, cellular, and microbiota approaches, along with potential mechanistic frameworks. One of the work’s features is its coherently defined structure. By categorizing and linking the various biomarkers to specific processes, the authors have demonstrated the interconnectivity between the defined cellular signaling pathways (Wnt/β-catenin, Hippo, Notch, PI3K/AKT, MAPK, JAK/STAT, TGF-β) and lymph node metastasis (LNM) relevant phenomena, namely, epithelial–mesenchymal transition, cell motility, and lymphangiogenesis. Such structure fosters the situational understanding that there is a functional mechanism of metastasis associated with the molecules that signify LNM. Including the LNM associated CTCs, the pivot to altered microbiome, and non-coding RNAs stretches investigative imagination beyond the confines of the tumor tissue (Wu et al.). The use of clinically functional biomarkers remains a challenging endeavor. Many LNM biomarkers are associated with primary research that is small in sample size, and are not externally validated. It would help to clarify the different tiers of evidence (discovery, retrospective validation, prospective validation) in the review, and to discuss pragmatic evidence limitations, such as assay reproducibility, sampling bias, cost, and imaging workflow integration. A more thorough examination of tumor microenvironment factors (immune and stromal remodeling, lymphatic vessel density) would help describe the portrait of metastasis beyond cancer-intrinsic signaling. Going forward, the field must prioritize prospective, multicenter validation of the leading biomarker candidates, ideally alongside imaging and clinicopathologic data to develop integrated multimodal prediction models. There is considerable untapped potential in spatial transcriptomics and serial liquid biopsies, particularly in cfDNA methylation and exosomal cargo. Functional validation of the biomarkers, i.e., demonstrating that their manipulation alters the metastasis process, will help eliminate the more trivial candidate biomarkers. Finally, translation will require thorough standard operating procedures, coordination across multiple jurisdictions, clinical utility studies, and, most importantly, evidence that biomarker-informed staging improves outcomes (Wu et al.). Wu et al. have set forth a comprehensive agenda. It is now the responsibility of the research community to deliver on this agenda and translate the molecular promise into precision staging that will direct treatment and improve survival in esophageal cancer.

Lung enteric-type adenocarcinoma (ETAC) is an extremely uncommon variant of non-small cell lung cancer (NSCLC), constituting less than 1% of all cases. The defining feature—intestinal differentiation within the lung complicates diagnosis and raises questions about its origin, molecular mechanisms, and treatment vulnerabilities. This case presents a unique ETAC with gastric metastases, marking the first reported instance of this metastatic pattern. The 63-year-old patient first underwent resection of a left lower lobe tumor with an intestinal immunophenotype (CDX2+, CK20+, Villin+) and absence of pulmonary markers (TTF-1−, NapsinA−). Almost 2 years later, she experienced a recurrence affecting the bronchus, bone, and significantly, the stomach sinus. A very common NRAS exon three mutation among lesions was confirmed by pathologic and molecular investigations, suggesting hematogenous spread from the original lung tumor. Following a partial response to platinum-based chemotherapy and the PD-1 inhibitor sintilimab, the patient’s progression-free survival was only 8 months (Li et al.). Three conclusions can be formulated from the current situation. The first of these underscores the difficulty of diagnosing ETAC, since it can mimic metastatic gastrointestinal cancer, therefore highlighting the need for molecular profiling accompanied by an in-depth histological examination. The biological behavior of ETAC also teaches us a great deal after finding stomach metastases and helps us understand the importance of finding non-traditional metastatic pathways. As shown by the clinical response to chemo-immunotherapy, the guided use of molecular and PD-L1 profiling, and immune checkpoint inhibitors, even in rare histologies, can be truly beneficial. This report provides a foundational understanding of ETAC’s pathobiology and its clinical management. Future work on the genomics and immunology of other cases will help define the connections between enteric differentiation, metastatic tropism, and the response to immunotherapy. Ongoing documentation of cases will aid in turning clinical anecdotes into evidence-based practice to support the precise management of rare variants in lung cancer (Li et al.).

In the past few years, the application of nanotechnology in cancer therapies has gained significant attention. The ability to control surface modifications to achieve specific binding to cancer cells has shown promise in the design of novel anti-cancer therapies. Mishra et al. have demonstrated the efficacy and safety of gold nanoparticles produced via green synthesis, an eco-friendly methodology that minimizes hazardous byproducts. The authors employed Bakuchiol isolated from the seed of *Psoralea corylifolia,* as a reducing and capping agent, demonstrating superior physicochemical properties and anticancer properties. The study establishes a promising technological advancement in the synthesis of safer nano-drug carriers for future targeted therapies. Although the physiological interaction of Bakuchiol-coated green nanoparticle further needs to be evaluated in preclinical models (Mishra et al.).

Taken together, this Research Topic highlights the importance of different aspects of tumor metastasis. The studies have contributed to elevating the understanding of the intricate molecular mechanisms that not only play a crucial role in the metastatic process but also provide the window for therapeutic interventions.

